# Exercise on Prescription. Effect of attendance on participants' psychological factors in a Danish version of Exercise on Prescription: A Study Protocol

**DOI:** 10.1186/1472-6963-8-139

**Published:** 2008-06-26

**Authors:** Thomas VG Bredahl, Lis Puggaard, Kirsten K Roessler

**Affiliations:** 1 Centre for Sports, Health and Civil Society, Institute of Sports Science and Clinical Biomechanics, University of Southern Denmark, Campusvej 55, DK-5230 Odense M, Denmark; 2 Institute of Sports Science and Clinical Biomechanics, University of Southern Denmark, Campusvej 55, DK-5230 Odense M, Denmark; 3 COWI A/S, Parallelvej 2, 2800 KOngens Lyngby, Denmark

## Abstract

**Background:**

In many countries exercise prescriptions are used to facilitate physical activity in a sedentary population with or in risk of developing lifestyle diseases. Some studies show a positive effect of exercise prescription on specific lifestyle diseases. Others only show moderately positive or no effect on physical activity level. Furthermore, the challenge is adherence of participants to a physically active lifestyle on a long term basis after intervention. Therefore, it is essential for offering successful prescribed interventions aiming towards behaviour change to focus on psychological and social issues as well as physiological issues. The aim of this study is to assess the short and long term development of psychological conditions in two different Exercise on Prescription groups; The Treatment Perspective and The Preventive Perspective behaviour. Thus, the aim of this paper is to describe the design used.

**Methods/Design:**

The Treatment Perspective involves a 16 week supervised training intervention including motivational counselling. The Preventive Perspective only involves motivational counselling. The study is an evaluation of best practice and is accomplished by the use of a combination of quantitative (collected by questionnaires) and qualitative (collected by the use of semi structured interviews) measures. Comparison of The Treatment Perspective and The Preventive Perspective are performed at baseline and after 16 months. Development within the groups is measured at 4, 10, and 16 months. Self-reported measures describe physical activity, health-related quality of life, compliance with national guidelines for physical activity, physical fitness, self-efficacy, readiness to change, decisional balance, and processes of change. To elaborate self-efficacy, readiness to change, decisional balance, and processes of change, these issues were elucidated by interviews.

**Discussion:**

This study of best practice is designed to provide information about important psychological concepts in relation to behaviour change and physical activity. The study is part of a health technology assessment of Exercise on Prescription, which apart from the psychological concepts (the patient's perspective) covers the effectiveness, the organization, and the health economy.

## Background

Studies have demonstrated that sedentary lifestyle is related to increased risk of lifestyle diseases [1-3]. Other studies have demonstrated a decreased risk of lifestyle diseases with an increased level of physical activity [4] and aerobic fitness [5]. Physical activity is recommended as treatment as well as prevention with regard to a number of lifestyle diseases [1,2]. Despite of increasing knowledge concerning benefits of physical activity, an increasing number of people are finding it difficult to meet the amount of health beneficial physical activity [6]. Approximately half of the Danish population is not sufficiently active [7] and studies show the same tendency for the Dutch and the American population [8-10]. This high percentage of inactive people is posing a serious threat to public health [11].

Interventions have started to accommodate the increasing demand of a physically active lifestyle. In many countries exercise prescriptions are used to facilitate physical activity in a sedentary population with or in risk of developing lifestyle diseases [12-14]. Different methods have been used to promote physical activity such as oral advice from the General Practitioner (GP), phone based counselling [12] and oral counselling from an exercise specialist [15] in combination with a low intensity physical activity intervention [13]. Studies of this type of intervention show contradictory results. Some studies show a positive effect on specific lifestyle diseases [16,17]. But reviews show that exercise prescriptions only have a moderately positive [18] or no [19] effect on physical activity level. Furthermore, research questions the effect of exercise prescriptions on a population level [20]. However, this kind of intervention is still widely used to promote physical activity [21]. Summarizing, a large percentage of participants stop being physical active after intervention even though the aim and future perspective are adherence.

In recognition of the difficulty in keeping the individual in a physically active lifestyle, Health Psychological research emphasize the fact that behaviour change is anchored in a psychological, social and physiological context [22-24]. In conclusion, it is essential for offering successful prescribed interventions aiming at behaviour change that attention is directed towards psychological and social issues as well as physiological [24]. The importance of the interaction between these factors is underlined by health psychological research showing that these factors in combination are influencing the individual health status and the ability and will to change behaviour [25-29].

Research have shown important factors with impact on behaviour change in relation to physical activity [28-30]. Due to a combination of the "Theory of Planned Behaviour" [30], "The Social Cognitive Theory" [28] and "The Trans Theoretical Model" [29], motivation, self-efficacy and readiness to change stand out as central concepts [10,31-35]. Even with this insight there is still a lack of knowledge about which and how these factors influence the individual trying to change behaviour during and after Exercise on Prescription (EoP).

It is important to recognize the significance of knowledge of the individual psychological precondition prior to inclusion into an exercise intervention. Likewise it is important to document changes in psychological conditions during and following the intervention. It is possible that the psychological precondition and development is just as important as the intervention itself in terms of gaining long term effect and persistence in a physically active lifestyle.

### Intervention

An exercise prescribed intervention used in Denmark in the County of Funen and the Municipality of Frederiksberg called EoP is used to initiate physical activity among sedentary individuals with or in risk of lifestyle diseases. EoP is divided into two areas.

**Figure 1:**
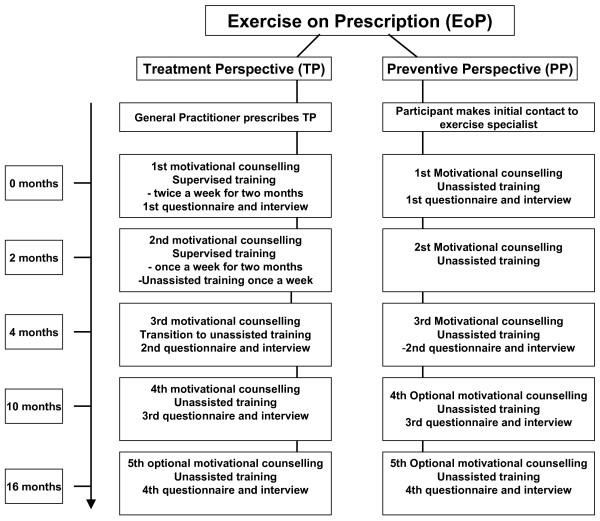
**Schematic overview of Exercise on Prescription in the County of Funen and Municipality of Frederiksberg**. Schematic overview of the two groups: The Treatment Perspective (TP) and the Preventive Perspective (PP) in Exercise on Prescription (EoP). In TP the general practitioner (GP) prescribes EoP for sedentary individuals with medically controlled conditions. The individual takes the prescription to a physiotherapist or an exercise specialist working with EoP. The participants complete four months of supervised training and motivational counselling. Questionnaires and interviewing are completed after 0, 4, 10 and 16 months. In PP the participant contacts the physiotherapist or exercise specialist working with EoP. The participants are included to PP if they are sedentary and in risk of developing lifestyle diseases that can be positively influenced by physical activity. The participants carry out unassisted exercise and receive motivational counselling at 0, 4, 10 and 16 month. Questionnaires and interviewing are completed after 0, 4, 10 and 16 months.

1) A Treatment Perspective (TP) directed towards individuals with specific medically controlled lifestyle diseases. The intervention design for TP was organised as described by Sørensen and colleagues [13]. The participants in TP were referred by their GP and followed a supervised group-based training intervention along with 8–12 other TP participants (including participants not taking part in the study) (Figure 1). The group-based supervised training was carried out by physiotherapists or exercise specialists. During the first two months, two weekly 1-hour training sessions were completed. During the final two months, one weekly training session was completed, supplemented by one weekly unassisted training session. The group-based training sessions involved elements of aerobic exercise (e.g. Nordic Walking, Aerobic), strength training, stretching and games. Furthermore, the participants were introduced to activities in the local area during the 4 months. The physiotherapist or the exercise specialist focused primarily on training improving aerobic capacity (Figure 1) [13]. In addition, the participants received motivational counselling at baseline and after four months. Subsequently they received voluntary phone based and/or personal motivational counselling after ten and sixteen months. The motivational counselling was carried out by physiotherapists or exercise specialists and used for making a physical activity schedule [36]. The motivational counselling aimed at increasing daily physical activity and concerned barriers towards being physically active and physical activity initiatives in the local area of the participant. The participant was responsible for carrying out the schedule.

2) A Preventive Perspective (PP) directed towards healthy citizens in risk of developing lifestyle diseases due to physical inactivity was conducted. The participants in PP entered the intervention by their own initiative. In PP, only the motivational counselling existed as a structured part of the intervention. It was carried out by physiotherapists or exercise specialists. Training was carried out unassisted or in existing local sports clubs or initiatives. Participants in PP received personal motivational counselling at baseline and after four months. Subsequently they received voluntary phone based and/or personal motivational counselling after ten and sixteen months. Further needs for counselling outside set time schedule were accommodated. The motivational counselling was used for making a physical activity schedule in co-operation between the physiotherapist or the exercise specialist and the participant [36]. The motivational counselling aimed at increasing daily physical activity and concerned barriers towards being physically active and physical activity initiatives in the local area of the participant. The participant was responsible for carrying out the schedule (Figure 1).

### Hypotheses

a) The participants' precondition in motivation, self-efficacy and readiness to change before the intervention is important for compliance. A psychological precondition to a greater extend directed towards behaviour change leads to a higher level of persistency in physical activity after intervention.

b) Participation in EoP leads to a positive change in motivation, self-efficacy and readiness to change. This positive change will lead to better adherence.

c) The participants' motivation, self-efficacy and readiness to change are different for TP and PP. Because PP participants enter the intervention by their own initiative, their psychological precondition is to a higher degree than the TP group expected to be directed towards behaviour change. This difference could be central for the participants' persistency in physical activity.

### Aim

The aim of this study is to assess differences between TP and PP concerning motivation, self-efficacy, readiness to change and self-reported physical activity. Moreover, to assess psychological differences within the groups in short (4 months) and long term (10 and 16 months) motivation, self-efficacy, readiness to change. Furthermore, the aim is to assess self-reported physical activity at the given time-points.

## Methods

The EoP intervention is evaluated as best practice to find out which of TP and PP has the greatest effect on the participants. Best practice is understood as the process of planning and organising the most appropriate intervention for the setting and population rather than as a gold standard or a packaged intervention [37,38]. EoP was evaluated from November 2005 until May 2008. The EoP intervention was launched 6 months prior to the study and offered by the County of Funen and the Municipality of Frederiksberg.

### Inclusion and exclusion criteria

GP's referred participants with the following characteristics to TP: 1) medically controlled lifestyle diseases (e.g. Type-2 diabetes and high blood pressure) 2) motivated to change lifestyle (this was estimated through personal conversation), 3) believed to be able to improve health from an increased level of physical activity, 4) willing to pay 750 Dkr. (100 €) for the intervention. At the PP intervention the participant could be advised to join PP by their general practitioner if they were physically inactive and/or did not meet any medically controlled condition to be enrolled in the TP. Furthermore, the participants could be enrolled by their own initiative by directly contacting the physiotherapist or exercise specialist. Information about PP was available at pharmacies, local media and health organizations (e.g. Diabetes Society, Heart Society, and The Danish Cancer Society).

In addition, GP's giving advice about TP and PP informed the participant about benefits of a physically active lifestyle in general.

### Recruitment

After referral to TP, about half of the patients receiving a prescription contacted the physiotherapist and/or the exercise specialist to make an initial appointment. All patients who made this initial appointment were entitled to be a part of the intervention.

All individuals contacting the physiotherapist and/or the exercise specialist in PP and complying with the inclusion criteria were permitted to be a part of the intervention.

Recruitment in both interventions took place over 21 months in 2005 to 2007. Informed consent was obtained.

### Outcome measures

The analyses in the study are performed as a combination of a quantitative and a qualitative method. The quantitative data are collected through questionnaires. The qualitative data are collected through semi structured qualitative interviews with key informants [39].

Information about psychological values and self-reported measures (BMI, physical activity, health-related quality of life, compliance with the national guidelines for physical activity, self-reported physical condition and psychological conditions towards behaviour change) are assessed at baseline and at the time of motivational counselling (Figure 1). Elaborating information concerning psychological concepts is gathered by interviews at the same time.

### Questionnaires

All self-reported measures were assessed by self-administrated questionnaires distributed by the physiotherapist and/or the exercise specialist at baseline and after four months. After ten and sixteen months the questionnaires are distributed by the author. All questionnaires are returned to the author by mail in pre-paid envelopes. Information is collected in the following four categories:

#### 1) Socio demographic information

Educational level, personal, and household income was assessed by asking e.g.: "Which kind of school education du you have?" "Do you have completed an education/vocational education?", "Which kind?", "How large was your and your household income last year (before taxes and deductions)?"

#### 2) Psychological factors

*Health-related quality of life *was assessed by using both the SF-12v2 [40] and EQ-5D [41]

*Readiness to change *in relation to physical activity was assessed by a questionnaire [42]. E.g. "As far as I'm concerned, I don't need to exercise regularly", "I really think I should work on getting started with a regular exercise program in the next 6 months", and "I have started exercising regularly within the last 6 months.

*Self-efficacy *in relation to physical activity was assessed by a questionnaire [42,43]. E.g. "I feel convinced that I am able to exercise 3 times or more a week with a duration of at least 20 minutes at a time even though: "I am under a lot of stress", "I feel I don't have the time", "I have to exercise alone", "I don't have access to exercise equipment", "I am spending time with friends or family who do not exercise", and "It's raining or snowing".

*Decisional balance *in relation to physical activity was assessed by a questionnaire [44]. "How important are the following opinions in your decision to exercise or not to exercise"? E.g. "I would have more energy for my family and friends if I exercised regularly", "I would feel more comfortable with my body if I exercised regularly" and "Regular exercise would help me have a more positive outlook on life".

*Processes of change *in relation to physical activity was assessed by a questionnaire [45]. "Think of similar experiences you may be currently having or have had during the past month". E.g. "I read articles about exercise in an attempt to learn more about it", "I get upset when I realize that people I love would have better health if they exercised", and "I have a friend who encourages me to exercise when I don't feel up to it. I have someone who encourages me to exercise".

#### 3) Physical activity

*Physical activity *was assessed by using a questionnaire, allowing conversion to MET (metabolic rate during quiet sitting) values [46,47].

*Compliance with national guidelines for physical activity *was assessed by asking "on how many days during an average week are you physically active more than 30 minutes?"

*Self-reported physical fitness *was assessed by asking "how do you rate your present physical fitness?", "how do you rate your present physical fitness compared to four months ago?", and "how do you rate your present physical fitness compared to people your age?" [13]

#### 4) Basic physiological information

*Bodyweight and height *were assessed by asking "Please write your bodyweight in kilos" and "Please write your height in centimetres".

*BMI *was calculated by dividing bodyweight (kg) with height (m) squared.

### Sample size questionnaire

To analyse the hypothesis of the study, the questionnaire consist of different scoring instruments used in other studies. Therefore, an overall power calculation of the sample size needed to analyse the full questionnaire was not possible. Instead, power calculations were done for key scoring instruments to indicate the number of participants needed to detect valuable changes. To detect a 2 point difference in the SF-12 Health Survey PCS and MCS scales over time within one group it requires a sample size of 140. To detect a 5 point difference a sample size of 46 is needed [40]. To detect a small to moderate effect (0.4) of VAS and EQ-5D index, a sample size of 100 is needed [48]. Power calculations were performed to estimate the sample size needed to detect a difference for Physical Activity. Estimated sample size for two-sample comparison of means done by the "sampsi" command in Stata 9.0 with means of 38 and 40, power as 0.8 and standard deviation as sd1(4) sd2(4) was 63. Estimated sample size for one sample with repeated measures done by the "sampsi" command in Stata 9.0 with the same values was 43 [49]. Power calculations were done to estimate the sample size needed to detect a difference for Self-efficacy. Estimated sample size for one sample with repeated measures done by the "sampsi" command in Stata 9.0 with a mean of 57, alternative mean as 61, power as 0.9 and standard deviation as sd(17) was 190 [49]. Using these assumptions for the overall study it was decided to include a sample size of 190 in each group.

All participants from TP and PP who volunteered and signed a written consent were included in the questionnaire study.

### Interviews

To strengthen and elaborate on the results from the questionnaire, psychological issues were assessed in semi-structured in depth interviews as well. The participants were strategically selected as key informants [50]. All interviews were performed in the participants' home by the author. Interviews are transcribed verbatim based on predetermined rules of transcription decided between the author and the transcriber [39]. Coding and analysis are primarily deductive and based upon the theoretical framework and the hypotheses presented earlier in the protocol [50]. Agreement on coding categories and interpretation is reached from having the author and a colleague analyzing the same interviews followed by discussion and adjustment differences. All coding and analysis were done by the author.

### Sample size interviews

Strategically selected key informants were asked to take part in the interview study. Selection criteria (age, gender, project, and educational background), were to assure that information gathered was adequate to cover the broad range of participants in the two projects. Those who signed a written consent were included in the study. To gather in depth information from the participants the sample size in the qualitative study was for TP 4 and for PP 3. Each participant was interviewed at baseline and after 4, 10, and 16 months.

### Statistical analysis

The changes from 0–4, 0–10, and 0–16 months within the two groups separately will be assessed using paired t-test or Wilcoxon rank sum test depending on distribution [51].

Comparisons between the TP and PP groups at baseline, four, ten and sixteen months will be assessed by Mann-Whitney test analyses [51]. Furthermore comparisons between TP and PP will be made with changes from 0–4, 0–10, and 0–16 months.

Drop-out analyses will be carried out for 0–4, 0–10, and 0–16 months. Compliant and non-compliant participants will be compared in regards to different baseline values. The effect of drop-out will be compared with differences between the TP and PP group.

Statistical significance was set at p < 0.05.

### Approval

The Danish Data protection Agency registration number is 2005-41-5248. Due to the non-biological and non-treating perspective of the study no registration to the local ethics committee was needed. ClinicalTrials.gov registration number: NCT00594360.

### Validation studies

Validation of the self-administrated questionnaire was done in a similar EoP intervention in Denmark using the same questionnaire. The test-retest reliability of the self-administrated questionnaire proved reliable in terms of agreement percent [18].

## Discussion

This study is designed to provide information about the patient perspective and best practise of a Danish EoP intervention in influencing psychological conditions towards behaviour change. The study is a part of a health technology assessment of EoP which besides the patient perspective covers the effectiveness [13], the organization, and the health economy [52].

## Competing interests

The authors declare that they have no competing interests.

## Authors' contributions

TVGB drafted the manuscript. All authors read, commented, and approved the final version of the manuscript. LP obtained funds for the project. TVGB, KKR and LP developed the project.

## Pre-publication history

The pre-publication history for this paper can be accessed here:


